# The Interphase Gas-Solid Synthesis of Ammonium Alginate—The Comparison of Two Synthesis Methods and the Effect of Low Molecular Weight Electrolyte Presence

**DOI:** 10.3390/ma15124321

**Published:** 2022-06-18

**Authors:** Nina Tarzynska, Anna Bednarowicz, Ewelina Pabjanczyk-Wlazlo, Zbigniew Draczyński

**Affiliations:** Institute of Materials Science of Textiles and Polymer Composites, Lodz University of Technology, Zeromskiego 116, 90-924 Lodz, Poland; anna.bednarowicz@dokt.p.lodz.pl (A.B.); ewelina.pabjanczyk-wlazlo@p.lodz.pl (E.P.-W.); zbigniew.draczynski@p.lodz.pl (Z.D.)

**Keywords:** ammonium alginate, sodium alginate, bio-based polyelectrolytes, polysaccharides, intrinsic viscosity

## Abstract

This paper presents a method for the synthesis of ammonium alginate by interphase gas-solid reaction. It was confirmed by FTIR ATR spectroscopy analysis that a full substitution of acid groups by ammonium groups on the surface of powdered alginic acid was performed. Comparative studies on the properties of ammonium alginate solutions obtained by interphase reaction with those prepared by the classical method of dissolving alginic acid in an ammonia solution showed that the rheological properties of the solutions from these two derivatives do not differ significantly. Moreover, it was shown that aqueous solutions of ammonium alginate are more stable over time than solutions of sodium alginate. It was confirmed that ammonium alginate and sodium alginate are typical polyelectrolytes, as the addition of a low molecular weight electrolyte to their solutions resulted in a decrease in viscosity.

## 1. Introduction

Alginates are naturally-occurring polysaccharide copolymers, consisting of the residues of ß-D-mannuronic acid (M blocks) and α-L-guluronic acid (G blocks), linked together by glycosidic bonds. These blocks occur in different proportions and in different distributions along the chain [1,2,3]. The amount of M and G blocks determines the physicochemical properties of the biopolymer chain. A higher proportion of G blocks ensures the easiness of gel formation, while the combination of MG blocks with a predominance of M blocks increases the flexibility and elasticity of the chain [4,5,6]. Due to the presence of rigid six-membered sugar rings and the limited possibility of rotation by glycosidic bonds, alginates are considered stiff molecules [7]. Alginates occur in the cell walls of brown algae as calcium, magnesium, and sodium salts of alginic acid [8]. They have been isolated from the cell walls of various species of brown algae. In the plant, they are responsible for preventing drying, maintaining cell integrity, and ensuring mechanical strength. Alginates have a wide range of applications, mainly in the food and pharmaceutical industries, due to their ability to retain water, form gels, and stabilize emulsions [9,10,11]. They are also of interest in tissue engineering and biomedicine due to their characteristic properties such as non-toxicity, biodegradability, and biocompatibility [12,13,14,15].

Alginates are also successfully used to produce fibres; most commonly, water-soluble sodium alginate is used for this purpose [16]. However, it is difficult to achieve a high process efficiency because the concentrations of sodium alginate spinning solutions in the range of 4 to 10% have an apparent dynamic viscosity which is below 60 Pa·s [17]. Exceeding the range of the 10% concentration results in a significant increase in the apparent dynamic viscosity, which results in an unstable spinning process. In order to obtain a spinning solution of the desired physicochemical properties, the apparent dynamic viscosity should maintained between 30 and 60 Pa⋅s, which ensures stable fibre formation [18,19]. Viscosity is also related to temperature—an increase of one degree Celsius causes a decrease in viscosity of up to 2.5%. Additionally, an effect of the presence of auxiliary agents, such as cations, e.g., Na^+^ and K^+^, on the viscosity of the alginate solutions is observed [20]. The introduction of small amounts of salts such as NaCl or KCl to the alginate solution affects the reduction in solution viscosity, which shows that the effects of electrostatic interactions between these cations and alginate are an important parameter for determining the rheological parameters of solutions [21,22].

Sodium alginate, due to its chemical structure, in diluted aqueous solutions exhibits properties typical for polyelectrolytes [23,24]. Polyelectrolyte solutions consist of ionizable polymer molecules, which are characterized by having a charge in dissolved form. Their physical properties result from the balance between the hydrophobic interactions of polymer chains and electrostatic interactions of side polar groups [25]. Polyelectrolytes are best characterized by the analysis of diluted solutions. It is then possible to obtain information about the conformation of the chains or the properties of the solutions [26,27]. 

Alginate fibres can be classified into many types, based on their type of material: alginic acid, zinc alginate, copper alginate, sodium alginate, calcium alginate, or ammonium alginate. Nevertheless, calcium alginate—as well as calcium sodium alginate—are most commonly used for the production of alginate fibres and are formed by the wet spinning method [28,29]. In the case of ammonium alginate fibres, no sources are available on fibre forming using this method. Ammonium alginate has been used to make alginate/clay aerogel composites [6].

The objective of this study was to compare two methods of ammonium alginate synthesis—interphase gas-solid and dissolution synthesis—as well as to determine the effect of sodium chloride’s presence on the properties of solutions of ammonium alginates.

## 2. Materials and Methods

### 2.1. Materials

Sodium alginate was used in the form of a powder (Protanal LF 10/60 LS), as a commercial product of FMC Biopolymer with an intrinsic viscosity of 2.609 dL/g and a molecular weight 89,000 g/mol (data delivered by producer). The ammonia solution (AS: 25%) and hydrochloric acid (HCl: 35–38%) were purchased from ChemPUR at chemical purity for analysis, and pure silver nitrate (AgNO_3_) from COPD. 

### 2.2. ^1^H NMR Spectroscopy

In order to determine the content of mannuronic and guluronic blocks in the examined sodium alginate and their ratio, ^1^H NMR analysis was performed using AVANCE II PLUS (Bruker BioSpin, Rheinstetten, Germany), operating at the ^1^H frequency 700 MHz. In order to perform the study, the polymer in powder form was dissolved in D_2_O. All spectra were calibrated at 4.70 ppm from the water peak, which was used as a chemical shift standard.

### 2.3. Alginic Acid Preparation/Synthesis 

A total of 8 g of sodium alginate was treated in 104.5 mL 2% aqueous hydrochloric acid solution for 24 h at room temperature in a 150 mL flask and stirred with a mechanical stirrer for 24 h. Subsequently, the polymer was drained in a laboratory dryer at 40 °C to a constant weight, and the reaction process was repeated 2 times. Then, the obtained alginic acid was rinsed several times with distilled water to obtain a neutral pH and prevent the reaction of the filtrate with AgNO_3_- residual hydrochloric acid must be removed to prevent degradation of the product obtained. Then, the product was dried at 60 °C to a constant weight. The alginic acid thus prepared was used for the synthesis of ammonium alginate. 

### 2.4. Synthesis of Ammonium Alginate

Ammonium alginate was obtained by two methods. In the first method, interphase gas-solid synthesis, alginic acid in powder form was placed in a desiccator over an open flat container containing a 25% aqueous ammonia solution (double molar excess in relation to carboxyl groups -COOH). The samples were kept over the ammonia solution for 1, 3, 5, 48, 96, and 216 h. After the determined period of time, the samples were transferred to a vacuum desiccator and kept for 48 h in order to remove free ammonia adsorbed on the surface of the samples. These samples were assigned as AlgNH_3_.

The second method, dissolution synthesis, consisted of the dissolution of alginic acid in 25% aqueous ammonia containing a double molar excess of ammonia in relation to carboxyl groups -COOH, obtaining a dissolved form of ammonium alginate. These samples were assigned as AlgNH_4_OH.

### 2.5. FTIR ATR Analysis 

Samples of sodium alginate, alginic acid, and ammonium alginate were examined with FTIR ATR analysis in powder form to confirm changes in their chemical structure. The study was carried out with the Thermo Scientific Nicolet 6700 using a resolution of 4 nm; each spectrum presented an average of 64 scans according to the procedure described in [30]. 

### 2.6. Assessment of Alginic Acid Substitution by Ammonium Groups 

In order to determine the degree of alginic acid substitution with ammonium groups, conductivity titration was performed. Changes in the electrical conductivity of aqueous ammonium alginate solutions were analysed (0.2 g AlgNH_3_/ 100 cm^3^ H_2_O), using 0.1 M hydrochloric acid solution as a titrant. This method was used for interphase gas-solid synthesis.

### 2.7. UV–Vis Analysis 

Diluted solutions of ammonium alginate were analysed by UV–Vis spectroscopy to confirm the occurrence of intermolecular interactions between alginate macromolecules and NaCl. Water, or an aqueous solution containing the same amount of NaCl as the test sample, was used as a reference solution. The test was performed with the Jasco device, model V-670. 

### 2.8. Analysis of Intrinsic Viscosity 

Solutions of sodium alginate and ammonium alginate, with a concentration of 0.4% polymer, differing in sodium chloride content (0%, 1%, 3%, 5%, 7% m/m) were filtered using a Gooch crucible (porosity 4), and then their flow time through the capillary was measured using an Ubbelhode viscometer (Si Analytics GmbH, Mainz, Germany) with constant k = 0.04905 mm^2^/s^2^ at 25 °C. Based on these measurements, the intrinsic viscosity was determined using Huggins [31] Equation (1).

(1) $$\eta_{\infty }=\frac{\sqrt{2}}{c}\cdot \sqrt{\left(\frac{t}{t_{0}}-1\right)\cdot \ln\left(t-t_{0}\right)}$$

where c—percentage concentration (g/100 cm^3^), t—sample flow time (s), and t_0_—solvent flow time (s). 

Hermans [32] suggested the following dependence of intrinsic viscosity on salt concentration (additional low molecular weight electrolytes) [32,33,34,35,36]:

(2) $$\eta =\eta_{\infty }+{\mathrm{SC}}_{s}^{-1/2}$$

where $\eta_{\infty }$—intrinsic viscosity, S—stiffness of the molecule, and C_s_—salt concentration.

A 20% sodium chloride solution containing 100 g of NaCl in 500 mL of water was prepared, which was then diluted to obtain solutions containing 1%, 3%, 5%, and 7% sodium chloride. 

### 2.9. Dynamic Lights Scattering (DLS)

Sodium alginate and ammonium alginate solutions with the addition of NaCl were characterized by the intensity weighted mean hydrodynamic size of the ensemble collection of particles (Z-Ave) and the polydispersity index (PDI). A ZetaSizer ZS (Malvern Instruments, Worcestershire, UK) instrument was used for the measurement procedure, and the applied temperature was 25 ± 0.1 °C. Solutions with a polymer concentration of 0.4 g/100 cm^3^ were tested in glass cuvettes. Each solution was tested 3 times.

## 3. Results

**Figure 1:**
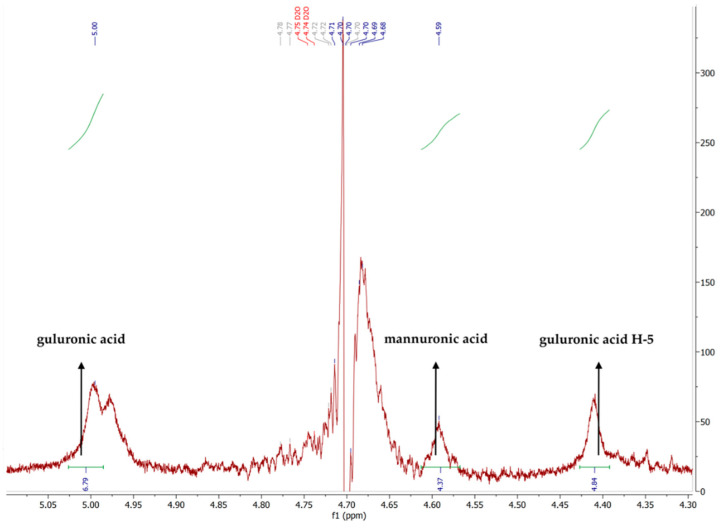
A fragment of the ^1^H NMR spectra of sodium alginate Protanal LF 10/60 LS.

^1^H NMR spectroscopy provided information on the content of mannuronic and guluronic blocks in the examined sodium alginate and their ratio [37]. The ^1^H NMR spectra of the anomeric region of sodium alginate Protanal LF 10/60 LS are given in Figure 1.

In the ^1^H NMR spectra of sodium alginate, the characteristic peaks were assigned to protons derived from mannuronic acid and guluronic acid groups. The peak at 5.00 ppm is attributed to the guluronic acid anomeric proton, at 4.59 ppm to the mannuronic acid anomeric proton, and at 4.42 ppm to guluronic acid H-5. As described by Grasdalen [38], from the relative areas of the three signals in this region, the mannuronic and guluronic ratio (M/G ratio) was calculated [38,39]. The molar fractions of these—mannuronic (FM) and guluronic (FG)—and the dyad are presented in Table 1.

Based on an analysis of the M/G ratio of the examined alginate, it can be concluded that its aqueous solutions form hard gels. Gels formed from alginate with a predominance of guluronic acid blocks are known as hard gels. When the alginate contains a predominance of mannuronic acid blocks, the transition to gel form is significantly easier. The formed gels appear to be soft gels, due to the increased water absorption and ability to exchange ions [40,41].

The FTIR ATR spectroscopy method was used to confirm the formation of the alginic acid and carboxylic group derivates obtained when carboxylic groups are substituted by Na^+^ and NH_4_^+^ cations.

**Figure 2:**
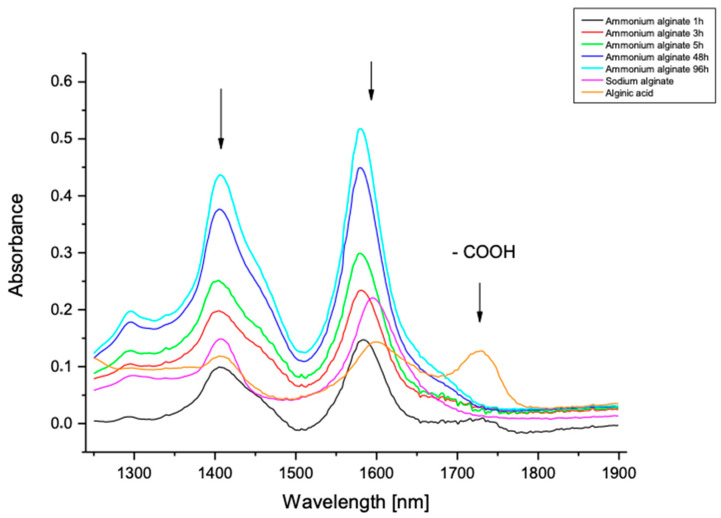
A fragment of the FTIR ATR spectrum of sodium alginate, alginic acid, and ammonium alginate, produced by the modification of alginic acid in an ammonia atmosphere in the selected conditions.

Figure 2 presents a comparison of the FTIR ATR spectra derived from ammonium alginate obtained by the modification of alginic acid in an ammonia atmosphere with different times of exposure (1, 3, 5, 48, and 96 h). As reference samples, the spectra of alginic acid and sodium alginate were taken. Based on this analysis, it is possible to observe changes in the spectra which are connected to the process of ammonium alginate salt formation. Firstly, we observe the disappearance of the characteristic bands of the carboxylic groups (-COOH) at a wavelength of 1725 nm, which confirms the substitution of the proton in the carboxylic group. Secondly, in the range of the signal stretching vibrations of the carboxylic bond C=O, shifts of the spectrum towards shorter wavelengths are observed. Thirdly, more intensive peaks appear with a maximum at 1580 nm, characteristic of a carboxylic group substituted with an ammonium group (-COONH_4_) [41,42,43,44]. Finally, there is a clear relation between the time of ammonium exposure and the intensity of the peak at 1580 nm. The higher exposure time, the higher level of substitution of the ammonium groups. 

In order to determine the degree of alginic acid neutralization with ammonium groups, a titration analysis of the obtained modified polymers was carried out. In the case of preparation of ammonium alginate by dissolution of alginic acid in an aqueous solution of ammonium, maximum substitution of carboxyl groups was assumed; thus, twice the molar excess of ammonia was used in relation to carboxyl groups. The results of this are presented in Table 2. 

On the basis of the analysis of the conductivity titration of ammonium alginate obtained as a result of the gas interaction of ammonia with alginic acid in the form of a powder, a linear relation can be observed. The increase in the exposure time to ammonia gas causes an increase in the degree of substitution with ammonium groups, which was also confirmed by the FTIR-ATR spectra (Figure 2). In the case of modifications carried out for 1, 3, and 5 h, slight differences in the substitution degree can be observed. A larger change can be observed no earlier than 48 h, but the most significant change is observed after 96 h. However, it can be observed that after 96 h of interaction, a maximum substitution of the available COOH acid groups with COONH_4_ groups is obtained, which means that using a longer exposure time has no benefit. If the treatment is applied for more than 96 h, no noticeable change is observed. 

In order to confirm the properties typical for the polyelectrolyte, i.e., changes in intrinsic viscosity values of solutions as a result of the presence of low-molecular weight electrolytes, comparative viscosity tests were carried out. The intrinsic viscosity of the prepared diluted solutions of sodium alginate and ammonium alginates obtained by the two methods was compared in the presence of NaCl. Table 3 presents the results of the intrinsic viscosity values of diluted sodium and ammonium solutions of alginic acid derivatives containing up to 1.2 mol/l NaCl. 

Based on the results presented in Table 3, a general significant decrease in the intrinsic viscosity value of the ammonium alginate solution in the presence of the NaCl can be observed, in the case of both synthesis methods, compared to commercially-used sodium alginate. 

A decrease of five, representing 6% of the intrinsic viscosity value of the ammonium alginate solution, is also visible in comparison with ammonium alginate obtained by dissolving alginic acid in ammonia solution while maintaining twice the excess of ammonium groups in relation to carboxyl groups. 

Additionally, the effect of solution ageing is visible, as an increase in the intrinsic viscosity value for all tested solutions of alginate without NaCl after a period of 216 h (9 days) from the preparation of the solution, as compared to measurements carried out within 48 h of the preparation of the solution. The highest values of intrinsic viscosity and the highest differences of its values after 216 h are observed for sodium alginate solutions despite the presence of NaCl. This is probably due to the facilitated formation of intermolecular bonds such as hydrogen bonds between sodium alginate macromolecules. Sodium alginate is the salt of a weak acid and a strong alkali; therefore, it undergoes a hydrolysis reaction, which results in the presence of undissociated COOH groups in the macromolecular chain. The presence of these groups in solutions of sodium alginate enables the formation of hydrogen intermolecular bonds. Ammonium alginate, unlike sodium alginate, which is the salt of a weak acid and a weak alkali, is predominantly present in an unhydrolyzed form. It can be expected that undissociated COONH_4_ groups in the side groups of ammonium alginate are present in the solution. The presence of these groups (COONH_4_) hinders the formation of intermolecular interactions in comparison with the COOH groups present in aqueous solutions of sodium alginate. 

On the basis of the analysis above, it can be concluded that the use of ammonium alginate is beneficial from the viewpoint of the technological process of wet fibre spinning because it allows for lower intrinsic viscosity values, enabling the use of higher polymer concentrations and influencing the efficiency of the spinning process. In addition, the use of ammonium alginate for this purpose is beneficial as it does not change the character of aqueous solutions over time. 

**Figure 3:**
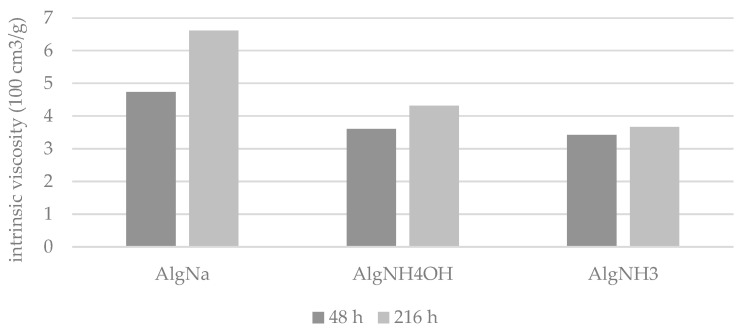
The comparison of the intrinsic viscosity values of ammonium alginates obtained by two methods in reference to aqueous solution of sodium alginate after 48 and 216 h.

Figure 3 presents a graphical comparison of the results of intrinsic viscosity measurements of solutions of sodium alginate (AlgNa), ammonium alginate obtained by the interaction of ammonia vapours with alginic acid (AlgNH_4_OH), and ammonium alginate solutions obtained by the dissolution of alginic acid in a double ammonia excess (AlgNH_3_). 

Based on an analysis of Figure 3, it can be observed that for all samples, an increase in the intrinsic viscosity value during storage occurs which is a natural phenomenon of the ageing of polymer solutions [45]. The highest tendency of increasing intrinsic viscosity after 216 h was observed for the sodium alginate solution—an increase of 40.42%. In ammonium alginate solutions, this relation is lower: for AlgNH_4_OH it is 19.4%, and for AlgNH_3_ it is the lowest, 8.8%. This confirms that the presence of the low molecular weight electrolytes can have a protective and anti-aging effect on the polymer solutions. 

**Figure 4:**
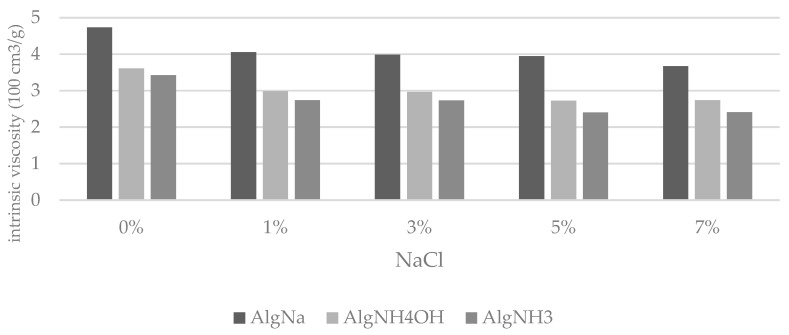
The comparison of intrinsic viscosity values of solutions depending on NaCl concentration after 48 h.

The next step included an analysis of the influence of the NaCl concentration in the solutions. In order to optimize the amount of salt ions introduced into the solutions of sodium alginate and ammonium alginate, solutions containing up to 7% NaCl were prepared. Taking into account the subsequent use of the ammonium alginate solutions as spinning solutions in the process of wet fibre formation, the intrinsic viscosity of the prepared solutions after 48 h storage at room temperature was measured. The results of the obtained values of intrinsic viscosity are shown in Figure 4. 

Based on the above analysis, it is possible to observe the existing difference in the intrinsic viscosity values between solutions of sodium alginate and ammonium alginates. These differences are likely to result from differences in the volume of polymeric clusters of sodium alginate and ammonium alginate in aqueous solutions. These derivatives, apart from differences in the size of Na+ and NH4+ cation substituents, also differ in their susceptibility to form intermolecular interactions such as hydrogen bonds. Sodium alginate has a higher susceptibility to this type of interaction compared to ammonium alginate. It was observed that NaCl addition at the level of 5% protects the macromolecules in solution from the formation of undesirable interactions between them, ensuring that even during longer storage of the solution, the intrinsic viscosity values do not change significantly. 

In order to confirm the occurrence of intermolecular interactions between ammonium alginate macromolecules and NaCl, a UV–Vis analysis of the solutions differing in salt ion content was performed. 

**Figure 5:**
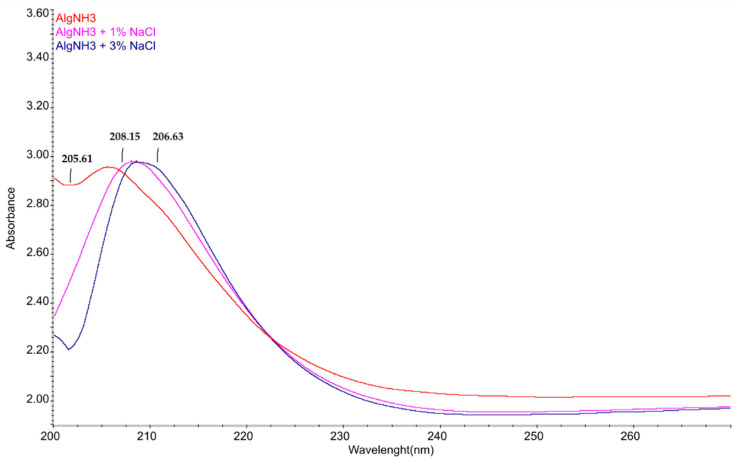
The UV–Vis spectra of the ammonium alginate, obtained as a result of ammonia vapour interaction with alginic acid.

Figure 5 shows an example of the UV–Vis spectra of ammonium alginate solutions in water containing 1% and 3% salt solutions. In the presented spectra, in comparison to the reference sample of ammonium alginate without additives, a shift in the signal of the carboxylic group of ammonium alginate in solution containing NaCl is visible, showing the occurrence of an interaction between them. 

Table 4 presents the size of particles and the polydispersity index of diluted solutions of sodium alginate and ammonium alginate in the presence of NaCl. The bonding of alkali metal ions, in this case Na^+^, to alginate is influenced by the electronegativity of the ion and the hydration radius, i.e., the electrostatic repulsion along the chain decreases as the hydration radius of the counterions increases. In the case of AlgNH_3_ and AlgNa, the addition of NaCl at 1% reduces the average particle size, while the PDI increases. However, the addition of 3% and a higher amount of NaCl increases the Z-Ave; this may be linked with the fact that in a solution containing a large fraction of small particles, these are surrounded by larger particles, which reduces the interactions with each other, leading to a reduction in the viscosity of the solution. In the case of solutions containing AlgNH_4_OH, where both NaCl and ammonia are the solvent, Z-Ave increases as the concentration of NaCl rises, while the PDI decreases. This is indicative of a lower polydispersity, meaning such solutions would be applicable in the wet-spinning process. However, the sudden increase in Z-Ave values, for both AlgNH_4_OH and AlgNH_3_, when the NaCl concentration exceeds 5% is presumably attributable to the precipitation of sodium alginate. 

By analysing the values of intrinsic viscosity and the hydrodynamic size of the ensemble collection of particles, it can be observed that in the case of AlgNa and AlgNH_3_, with an increase in NaCl concentration, a decrease in Z-Ave can be observed; this is due to the formation of agglomerates and the subsequent precipitation of sodium alginate.

## 4. Discussion

The results obtained throughout this study indicate that it is possible to obtain an alginic acid derivative as a result of the gas-solid interfacial reaction. Alginic acid is an excellent adsorbent of gaseous ammonia. The sorption capacity for powdered alginic acid reaches 73% of the presence of COOH groups. It could therefore be used in filters protecting against ammonia vapours.

During a comparison of the properties of ammonium alginate solutions obtained by the adsorption of ammonia gas with ammonium alginate and those obtained by dissolving alginic acid in an ammonia solution, no significant differences were found in the intrinsic viscosity values of the diluted solutions.

Solutions of ammonium alginate have lower intrinsic viscosities than solutions of commercially-used sodium alginate. This is probably a consequence of the fact that ammonium alginate is the salt of a weak acid and a weak alkali, and therefore does not undergo a hydrolysis reaction. The presence of undissociated groups of ammonium alginate causes a space-restriction which limits the formation of intermolecular bonds, such as hydrogen bonds, which can in turn affect the viscosity of the solution.

Solutions of ammonium alginate are characterized during preservation by a higher stability of viscosity parameters in relation to solutions of sodium alginate. In the case of ammonium alginate obtained as a result of an interphase reaction, after 9 days, an increase in viscosity value of about 8.8% is observed, which in the case of the analogous treatment of a sodium alginate solution results in an increase of 40.4%. 

The introduction of sodium chloride into solutions of sodium and ammonium alginate results in the formation of interactions between the alginate macromolecule and NaCl ions. The formation of these interactions is visible in the UV–Vis spectrum, as well as in the phenomenon of the decreasing intrinsic viscosity of the solution as the amount of introduced salt increases. The decrease in the viscosity of the solutions tested was observed until a salt concentration of 5% was obtained. Further increases in the salt content of the solution did not result in a more significant reduction in the viscosity value. Contrary to the use of salt solutions, an increase in viscosity during storage is observed in solutions without NaCl. Therefore, it can be concluded that the presence of low molecular weight electrolytes in polyelectrolyte solutions has protective properties against the formation of intermolecular interactions.

On the basis of the results obtained, it can also be concluded that the use of ammonium alginate, as a polymer which does not significantly change the character of aqueous solutions over time, is beneficial from the technological aspect of the wet-spinning process. 

## Figures and Tables

**Table 1:** The content of blocks G and M in the sodium alginate Protanal LF 10/60 LS.

F_M_	F_G_	M/G	F_MM_	F_MG_	F_GM_	F_GG_
0.29	0.71	0.41	0.09	0.20	0.20	0.51

Where: F_M_—molar fraction of mannuronic acid groups, F_G_—molar fraction of guluronic acid groups, M/G—mannuronic and guluronic ratio, F_MM_—homopolymeric mannuronic blocks, F_MG_/F_GM_—heteropolymeric fractions, F_GG_—homopolymeric guluronic blocks.

**Table 2:** Degree of alginic acid substitution by ammonium groups.

Treatment Time (h)	Degree of Alginic Acid Substitution by -COONH_4_ (%)
1	57.1
3	61.2
5	60.8
48	65.6
96	73.0
216	73.1

**Table 3:** The intrinsic viscosity values of diluted solutions of sodium alginate and ammonium alginate in the presence of NaCl.

		C_s_ (mol/l)				
		0	0.17 (1%)	0.51 (3%)	0.86 (5%)	1.20 (7%)
		Intrinsic Viscosity η (100 cm^3^/g)				
AlgNa	48 h	4.7 ± 0.2	4.2 ± 0.1	4.1 ± 0.1	4.0 ± 0.2	3.7 ± 0.1
	216 h	6.6 ± 0.1	4.5 ± 0.1	4.1 ± 0.1	3.8 ± 0.2	3.5 ± 0.1
AlgNH_4_OH	48 h	3.6 ± 0.2	3.1 ± 0.2	3.0 ± 0.2	2.8 ± 0.1	2.8 ± 0.2
	216 h	4.3 ± 0.3	3.5 ± 0.2	3.3 ± 0.1	3.1 ± 0.1	2.9 ± 0.1
AlgHNH_3_	48 h	3.4 ± 0.2	2.9 ± 0.2	2.8 ± 0.1	2.5 ± 0.2	2.5 ± 0.1
	216 h	3.7 ± 0.1	2.8 ± 0.1	2.7 ± 0.1	2.5 ± 0.1	2.3 ± 0.2

Where: AlgNa—sodium alginate, AlgNH_4_OH—ammonium alginate obtained by dissolution synthesis, AlgHNH_3_—ammonium alginate obtained by interphase gas-solid synthesis.

**Table 4:** The size of particles (Z-Ave) and the polydispersity index (PDI) of diluted solutions of sodium alginate and ammonium alginate in the presence of NaCl.

	AlgNH_4_OH			AlgNH_3_		AlgNa	
	NaCl	Z-Ave (nm)	PDI	Z-Ave (nm)	PDI	Z-Ave (nm)	PDI
24 h	0%	421.3	0.569	248.1	0.484	558.4	0.633
	1%	787.1	0.775	330.2	0.525	281.6	0.722
	3%	875.2	0.774	174.2	0.501	601.3	0.916
	5%	1361	0.232	416.2	0.840	771.7	0.972
	7%	1682	0.673	1014	0.751	849.3	0.923
48 h	0%	298	0.562	239.3	0.501	621	0.650
	1%	417.2	0.575	173.4	0.531	231.5	0.946
	3%	541.7	0.878	1684	0.929	547.2	0.874
	5%	2006	0.305	2265	0.678	356.1	0.788
	7%	2330	0.184	4489	0.578	529.5	0.774
216 h	0%	244.2	0.540	260	0.490	602.1	0.829
	1%	504	0.607	127.3	0.701	203.2	1
	3%	487.4	0.885	599.4	0.899	672.8	1
	5%	896.1	0.944	4555	0.617	1345	1
	7%	7063	0.342	5317	0.494	213.4	0.793

Where: Z-Ave—hydrodynamic size of the ensemble collection of particles, PDI—the polydispersity index.

## Data Availability

Data are contained within the article.
